# Supplementary figures and images for: Reprogramming feedback strength in gibberellin biosynthesis highlights conditional regulation by the circadian clock and carbon dioxide
Source: PLoS One. 2025 Dec 9;20(12):e0337439. doi: 10.1371/journal.pone.0337439 (PMC12688126; doi:10.1371/journal.pone.0337439)

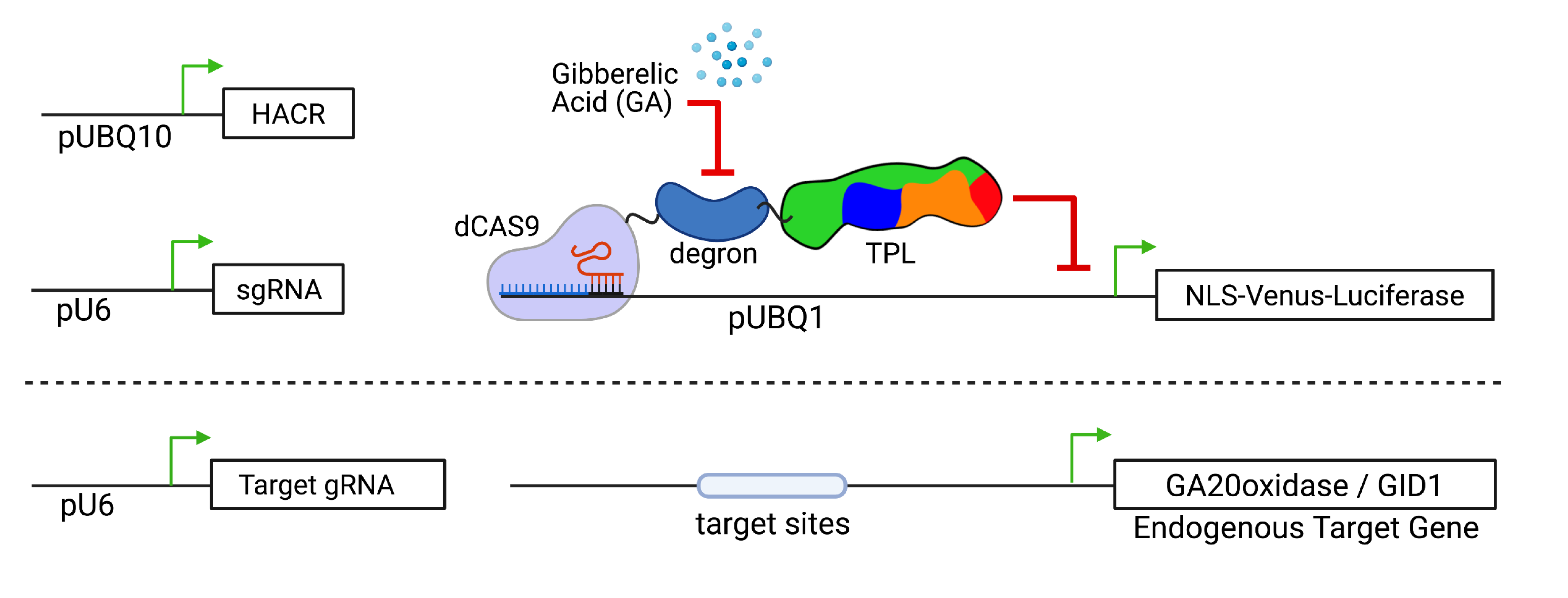

Supplement: S1 Fig — The top schematic of the genetic circuit used to build GA responsive HACRs (above dotted line) was described in [34]. In the lower portion of the schematic (below the dotted line) demonstrates the additional pAt-U6 driven gRNA which targets the endogenous genes (i.e., GA20ox or GID1). This allows the GA-HACR to simultaneously act not only as a reporter via Venus/Luciferase, but also to modify existing genetic networks. (TIF) [file pone.0337439.s001.tif]

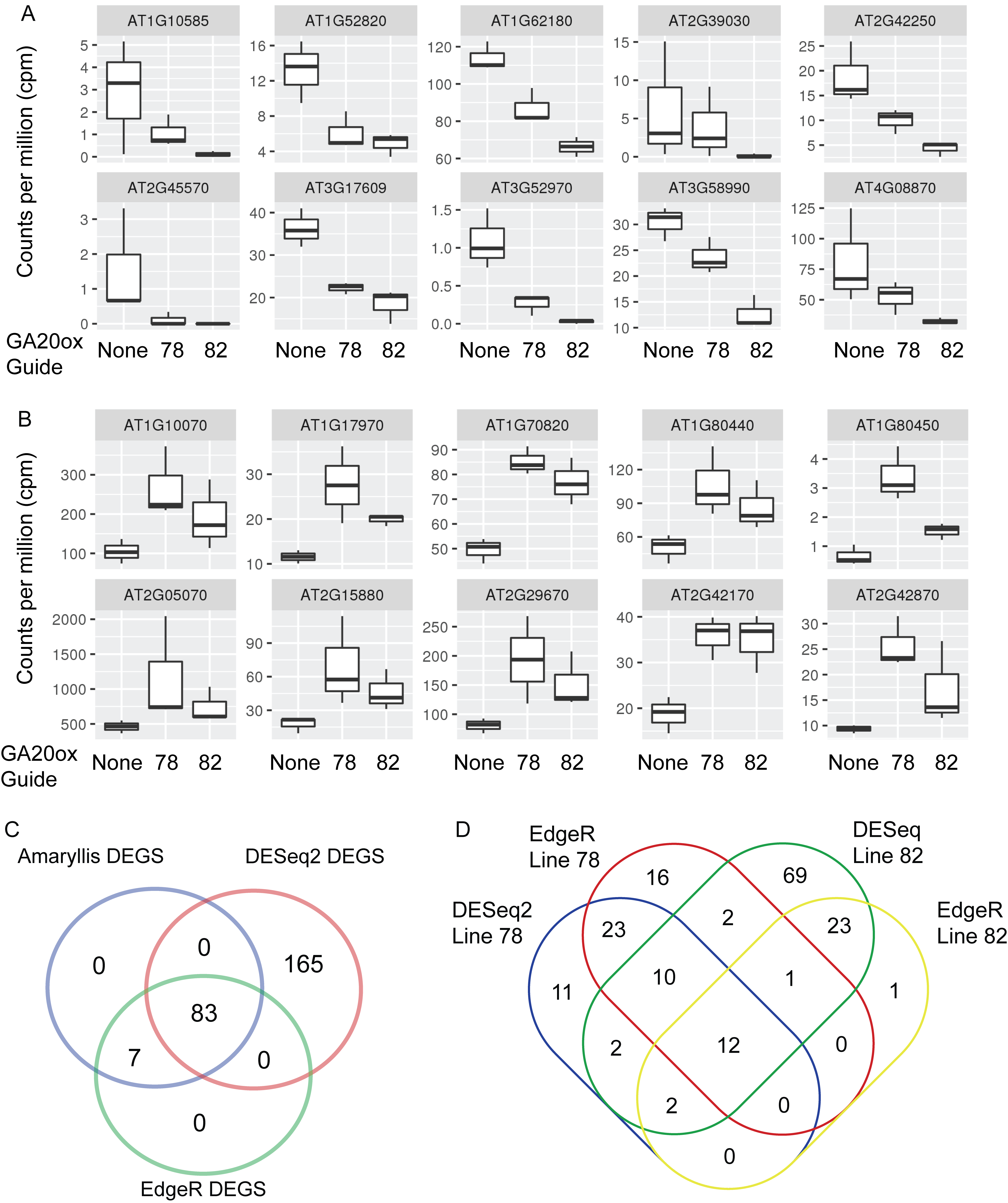

Supplement: S2 Fig — A-B. Selected DEGS were chosen to be graphed to demonstrate gene expression changes across the two selected lines (78 and 82) and wild type. We selected 10 down-regulated (A) and 10 up-regulated (B) DEGs, and graphed the counts per million across the 3 replicates in standard boxplots to demonstrate the similar trends across the two lines. C. To ensure that we were robustly detecting the maximum impact of the GA-HACR intervention, we applied two supplemental DEG finding packages, DESeq2 and EdgeR, and plotted the overlap in detected DEGs. D. A more detailed breakdown of the DEGs identified in the two DEG caller methods separated by genetic line. (TIF) [file pone.0337439.s002.tif]

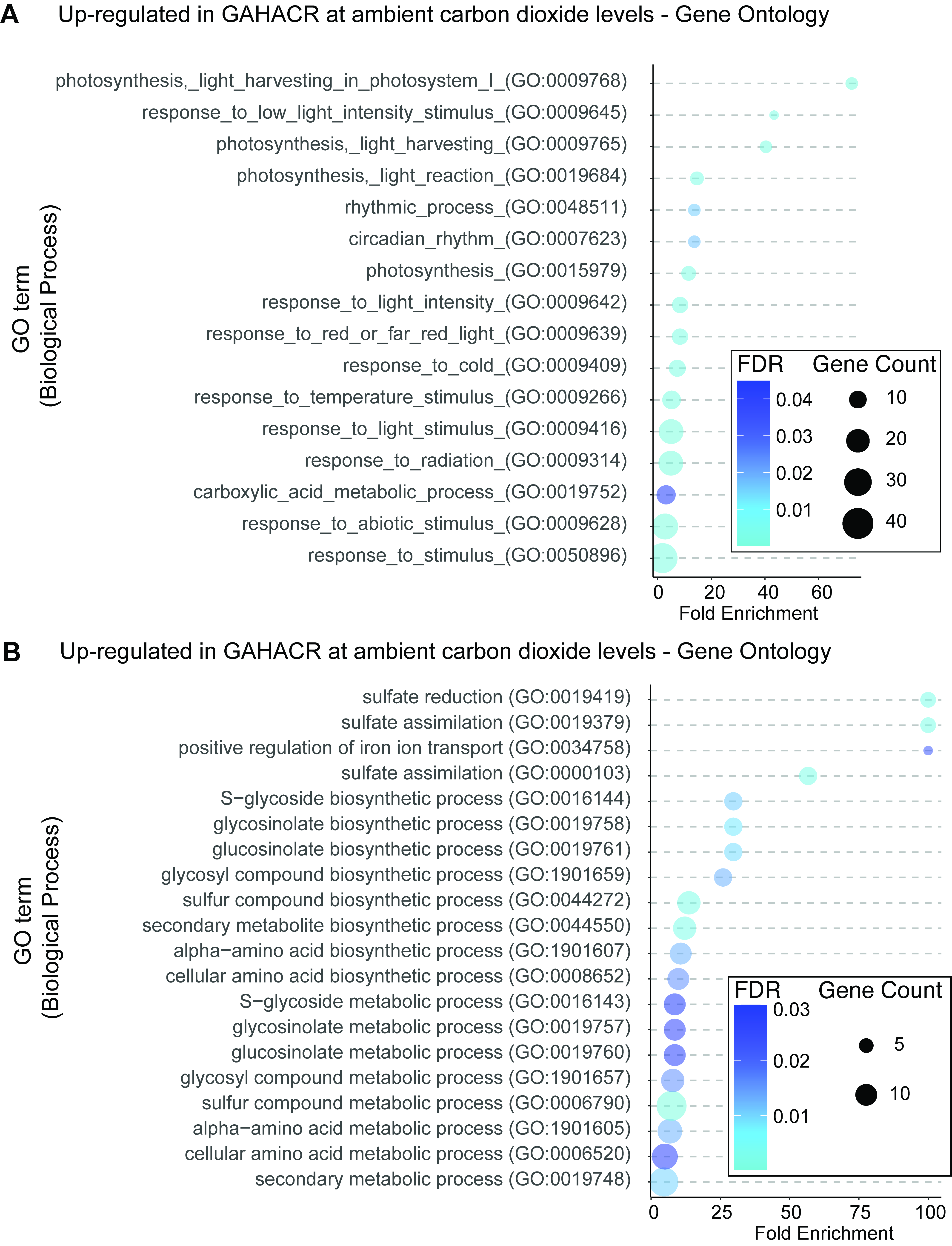

Supplement: S3 Fig — Gene Ontology terms enriched in the upregulated (A) and downregulated (B) differentially expressed genes from GAHACR targeted to GA20ox at ambient carbon dioxide levels. FDR – False discovery rate, GO was called via gProfiler. Data was graphed in R, using the ggplots2 package (see methods). (TIF) [file pone.0337439.s003.tif]

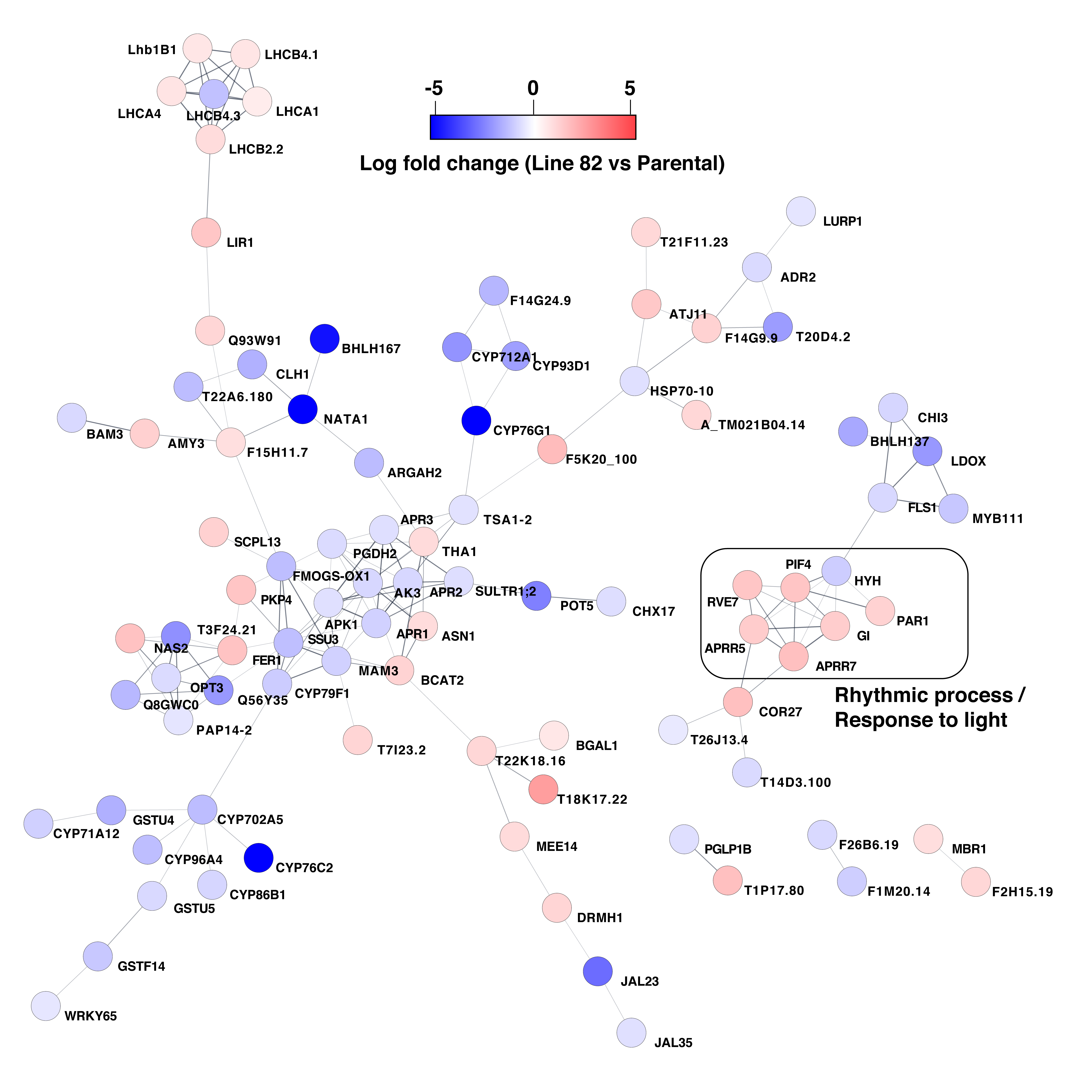

Supplement: S4 Fig — DEGs from the GA-HACR RNA-sequencing performed at ambient carbon dioxide levels were imported into Cytoscape for network analysis using the STRING database. All singletons were trimmed, and functional enrichment was performed. A subnetwork of Rhythmic processes and Light responsive proteins were identified and excerpted into Fig 3E. Nodes are color coded based on the log fold change observed in the GA-HACR lines 82 versus the parental GA-HACR line. DEGs that demonstrated a reduction in expression are colored blue and DEGs that increased expression are red (see scale). (TIF) [file pone.0337439.s004.tif]

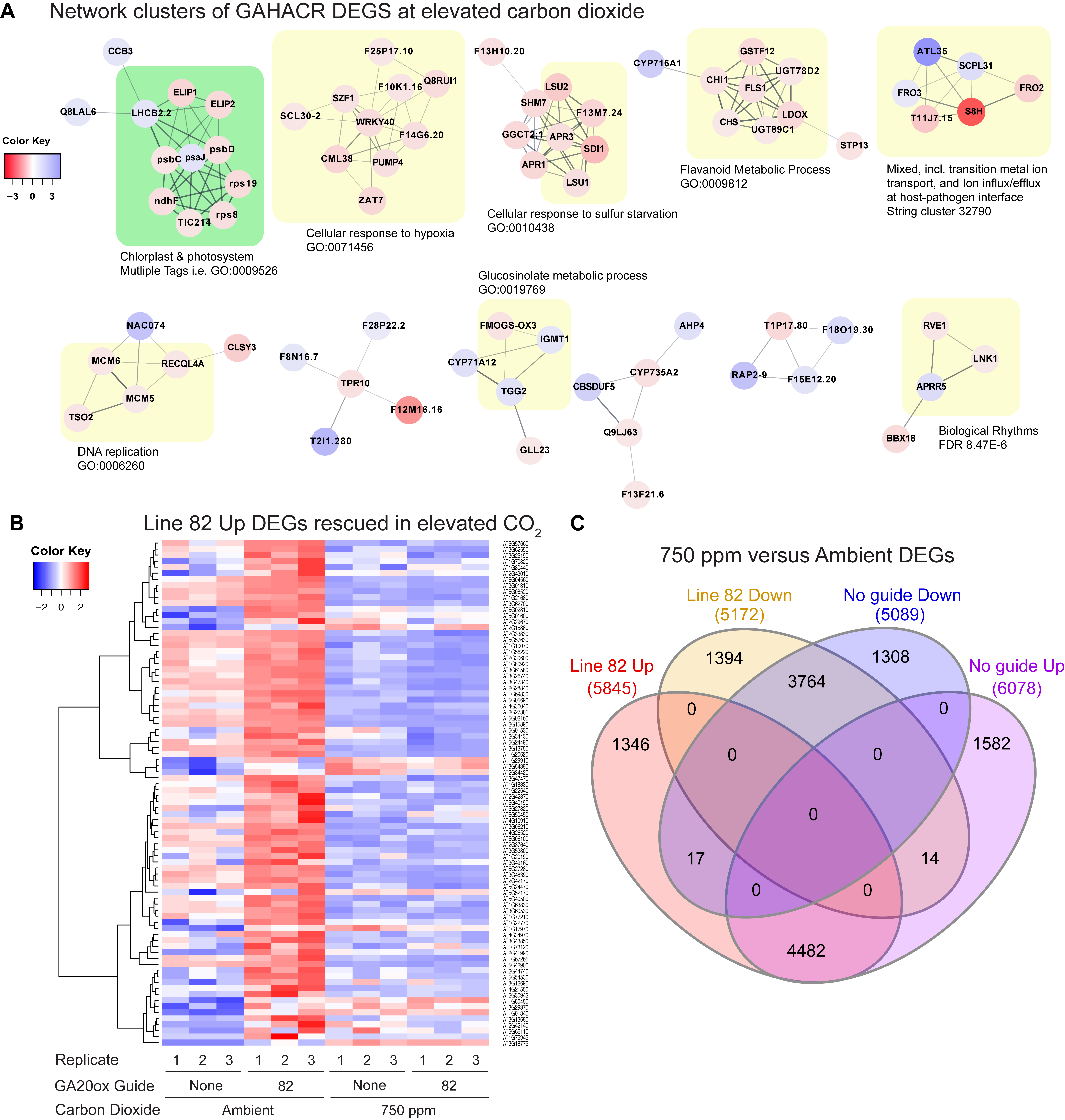

Supplement: S6 Fig — DEGs from the GA-HACR RNA-sequencing performed at elevated (750 ppm CO2) carbon dioxide levels were imported into Cytoscape for network analysis using the STRING database. All singletons were trimmed, and functional enrichment was performed. The network was clustered using MCL (inflation value = 4). A subnetwork of Rhythmic processes was identified (bottom right, biological rhythms) that includes the genes PRR5 and RVE1. Nodes are color coded based on the log fold change observed in the GA-HACR lines 82 versus the parental GA-HACR line. DEGs that demonstrated a reduction in expression are colored blue and DEGs that increased expression are red (see scale). B. Heatmap of top upregulated DEGs identified by RNA-seq analysis at ambient CO2 levels (left 6 columns). Only line 82 upregulated DEGs are shown for simplicity, and many appear to be reduced in expression at elevated carbon dioxide levels (right 6 columns). Values are in Log2, where red is upregulated, and blue is downregulated. C. Intersection of DEGs generated by comparing ambient to elevated carbon dioxide demonstrate a large number of genes that are differentially expressed regardless of the GAHACR intervention. (TIF) [file pone.0337439.s006.tif]

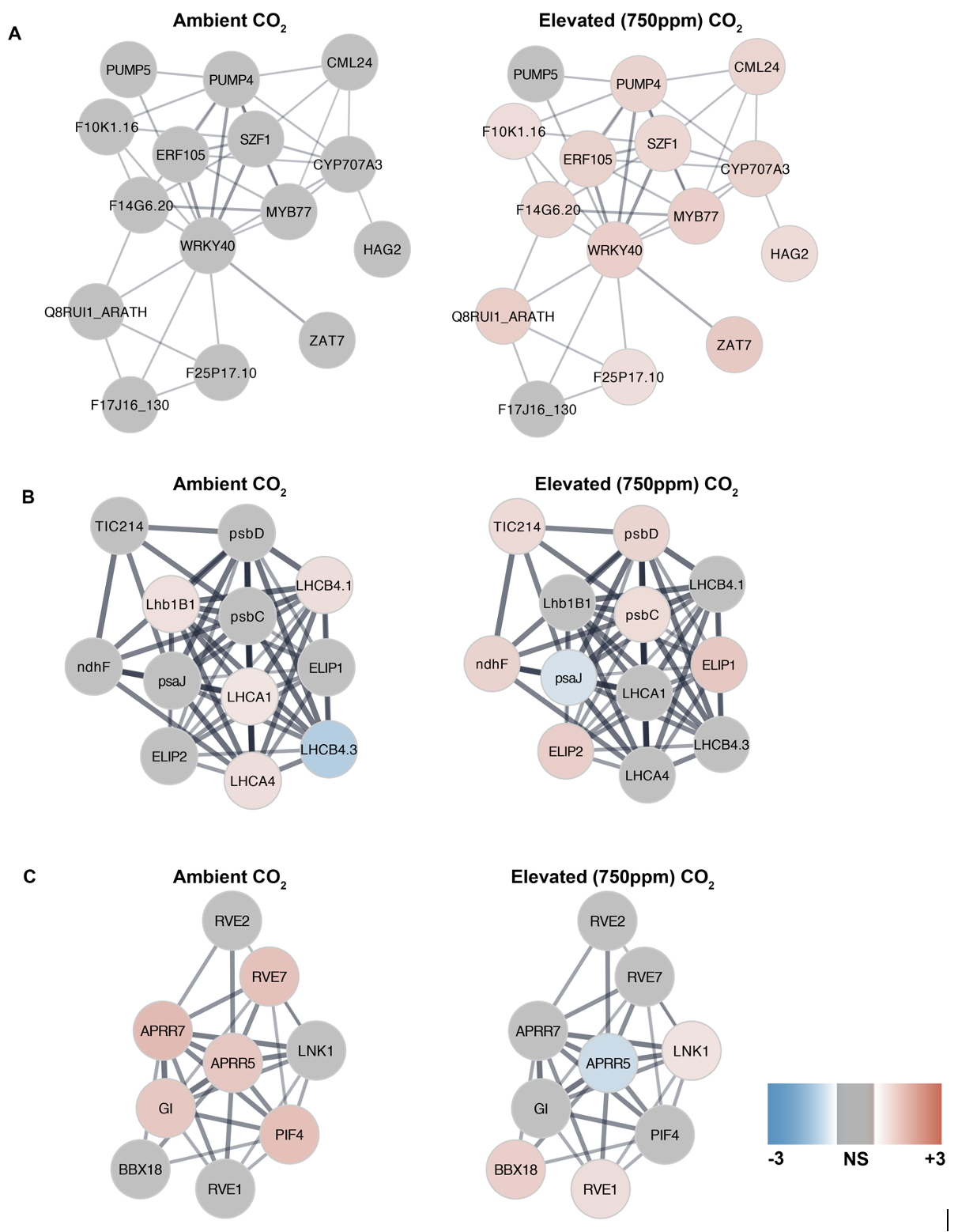

Supplement: S7 Fig — DEGs from the GA-HACR RNA-sequencing performed at ambient and elevated (750 ppm CO2) carbon dioxide levels were pooled and imported into Cytoscape for network analysis using the STRING database. All singletons were trimmed, and functional enrichment was performed. The network was clustered using MCL (inflation value = 4), and we manually subset selected clusters to examine how elevated carbon dioxide influences the networks. Each cluster node was colored based on the differential gene expression from the ambient (left column) or elevated (right column) no guide versus line 82 GAHACR experiments. A. Cluster defined by GO term Cellular response to hypoxia, GO:0071456, FDR = 1.02E-9. B. Cluster defined by GO Cellular component keyword Photosystem, GO:0009521, FDR = 3.12E-20 C. Cluster defined by UniProt keyword Biological Rhythms, KW-0090, FDR = 8.27E-16. (TIF) [file pone.0337439.s007.tif]
