# Supplementary material for: Reprogramming feedback strength in gibberellin biosynthesis highlights conditional regulation by the circadian clock and carbon dioxide
Source: PLoS One. 2025 Dec 9;20(12):e0337439. doi: 10.1371/journal.pone.0337439 (PMC12688126; doi:10.1371/journal.pone.0337439)
Supplement: S3 Table — Oligonucleotides used in this study. (PDF) [file pone.0337439.s010.pdf]

S3 Table. Primer Table

Primer Name	Sequence	Relates to Fig
PP2AA3 F	GCGGTTGTGGAGAACATGATACG	Fig 3F,J
PP2AA3 R	GAACCAAACACAATTCGTTGCTG	Fig 3F,J
CCA1 F	CCAGATAAGAAGTCACGCTCAGAA	Fig 3F
CCA1 R	GTCTAGCGCTTGACCCATAGCT	Fig 3F
GA20ox1_qF	CTCATGAATACACGAGCC	Fig 3F,J
GA20ox1_qR	TGATACACCTTCCCAAATG	Fig 3F,J
GA20ox2_qF	ATGCTCACCGTTTGATGG	Fig 3F,J
GA20ox2_qR	CCTTCCCAAACTGCTCG	Fig 3F,J
GA20ox3_qF	CCTATCTGCATATGGACTC	Fig 3F,J
GA20ox3_qR	AAACCTTCCCGAAATCTTC	Fig 3F,J
GA20ox5_qF1	ACAGGGCAGTGGTAAACAAG	Fig 3F,J
GA20ox5_qR1	TCACCTTCCACAAGTTCAGGAG	Fig 3F,J
GA20ox5_qF2	AGGGCAGTGGTAAACAAGGAG	Fig 3F,J
GA20ox5_qR2	AGCTGAGCCCAAGTAAAGTCAG	Fig 3F,J
GI F	TTGCAACTCCAAGTGCTACG	Fig 3F,J
GI R	GCTCGAAGGAGTTCCACAAG	Fig 3F,J
LUC_F	CGTCGCCAGTCAAGTAACAA	Fig 3J
LUC_R	TCGTCCACAAACACAACTCC	Fig 3J
PIF4 F	CCAGATCATCTCCGACCGGTTTG	Fig 3F,J
PIF4 R	CTAGTGGTCCAAACGAGAACCGT	Fig 3F,J
PRR7 F	CTGCACTCGTTATATCGTTACTG	Fig 3F,J
PRR7 R	GGCATGATCACCTCTGTTAG	Fig 3F,J
SAUR15_q_F	CAATGGGCGGCTTAACAATA	Fig 3J
SAUR15_q_R	TCTGAGATGTGACTGTGAAGAACA	Fig 3J
TOC1 F	CACAGGGATTCTGCGAAG	Fig 3F,J
TOC1 R	CTCTCCTTTCAGAGTGTTCTTATC	Fig 3F,J
