# Supplementary material for: Reprogramming feedback strength in gibberellin biosynthesis highlights conditional regulation by the circadian clock and carbon dioxide
Source: PLoS One. 2025 Dec 9;20(12):e0337439. doi: 10.1371/journal.pone.0337439 (PMC12688126; doi:10.1371/journal.pone.0337439)
Supplement: S8 Model Information — Gibberellin model from Middleton et. al. [24] with new terms utilized in this study. (PDF) [file pone.0337439.s011.pdf]

Supplemental Model Information.

Gibberellin perception.

GA_4 binds reversibly to GID1; bound GID1 undergoes a conformational change, whereby its lid closes (at rate *q*) and opens (at rate *p*):

$$\mathrm{GID1^o + GA_4} \underset{l_d}{\overset{l_a}{\rightleftharpoons}} \mathrm{GID1^o.GA_4}, \qquad \textbf{[1]}$$

$$\mathrm{GID1^o.GA_4} \underset{p}{\overset{q}{\rightleftharpoons}} \mathrm{GID1^c.GA_4}. \qquad \textbf{[2]}$$

Superscripts o and c indicate that the lid is open and closed, respectively.

$$\mathrm{GID1^c.GA_4 + DELLA} \underset{u_{d_1}}{\overset{u_{a_1}}{\rightleftharpoons}} \mathrm{GID1^c.GA_4.DELLA_1}, \qquad \textbf{[3]}$$

$$\mathrm{GID1^c.GA_4.DELLA_1} \xrightarrow{u_m} \mathrm{GID1^c.GA_4 + DELLA^*}, \qquad \textbf{[4]}$$

$$\mathrm{GID1^c.GA_4 + DELLA} \underset{u_{d_2}}{\overset{u_{a_2}}{\rightleftharpoons}} \mathrm{GID1^c.GA_4.DELLA_2}. \qquad \textbf{[5]}$$

Subscripts distinguish the two types of binding: 1 indicates the more stable binding, which mediates degradation of DELLA proteins, and 2 indicates the less stable form.

Gibberellin biosynthesis.

The gibberellin precursor, GA_{12}, is assumed to be produced at a constant rate ω_{GA12}. GA_{12} is converted to GA_{15}, then to GA_{24}, and finally, to GA_9, and each time, it is facilitated by members of the GA20ox family of enzymes:

$$\mathrm{GA_X + GA20ox} \underset{k_{d_X}}{\overset{k_{a_X}}{\rightleftharpoons}} \mathrm{GA_X.GA20ox} \xrightarrow{k_{m_X}} \mathrm{GA_Y + GA20ox\ fo[illegible]}$$

$$(\mathrm{X, Y}) = \{(12, 15), (15, 24), (24, 9)\}. \qquad \textbf{[6]}$$

In the final step of the biosynthesis pathway, GA3ox converts GA_9 to GA_4:

$$\mathrm{GA_9 + GA3ox} \underset{k_{d_9}}{\overset{k_{a_9}}{\rightleftharpoons}} \mathrm{GA_9.GA3ox} \xrightarrow{k_{m_9}} \mathrm{GA_4 + GA3ox}. \quad \textbf{[7]}$$

Exogenous GA₄.

Assumes that the rate of GA_4 transport across the cell membrane of a root is given by

$$P_{\mathrm{mem}} \frac{S_{\mathrm{root}}}{V_{\mathrm{root}}} \left(A_1 \omega_{\mathrm{GA4}} - B_1 \mathrm{GA}_4\right), \quad \textbf{[8]}$$

where ω_{GA4} is the externally applied concentration of GA_4, P_{mem} is the permeability of the membrane, S_{root} is the root surface area, V_{root} is the root volume, and A_1 and B_1 are the proportions of protonated GA_4 in the cell wall and cytoplasm, respectively. See Middleton et al., SI Appendix, B.1 Exogenous GA_4 for estimated values.

Gibberellin-mediated gene regulation.

We write $[X_m]$ for the concentration of an mRNA and $[X]$ for the corresponding protein. GA20ox, GA3ox and GID1 are up-regulated by DELLA protein, and therefore, the rate of mRNA transcription is an increasing function of $[\mathrm{DELLA}]$ that is balanced by degradation at a rate φ_X and normalized such that the maximum possible steady-state mRNA concentration is equal to one:

$$\frac{d[\mathrm{X_m}]}{dt} = \varphi_{\mathrm{X}} \left(\frac{[\mathrm{DELLA}]}{[\mathrm{DELLA}] + \theta_{\mathrm{X}}} - [\mathrm{X_m}] \right) \text{ for X} = \{\mathrm{GA20ox, GA3ox, GID1}\}. \quad \textbf{[9]}$$

Transcription of DELLA mRNA is repressed by DELLA protein, and therefore, we use the equivalent decreasing form of transcription rate:

$$\frac{d[\mathrm{DELLA_m}]}{dt} = \varphi_{\mathrm{DELLA}} \left(\frac{\theta_{\mathrm{DELLA}}}{[\mathrm{DELLA}] + \theta_{\mathrm{DELLA}}} - [\mathrm{DELLA_m}] \right). \quad \textbf{[10]}$$

In all cases, θ_X is the DELLA protein concentration for half-maximal transcription. We also assume that X_m is translated at a rate δ_X and that the gibberellin biosynthesis enzymes (GA20ox and GA3ox) and the gibberellin receptor (GID1) are degraded at a

constant rate μ_x. DELLA proteins are turned over by the mechanism described in Eqs. 3-5.

Engineered repression term (new term [11]).

To simulate the GAHACR's or No Degron CR's repression of GA20ox expression, we incorporated a term that captures GAHACR protein concentration scaled by a user defined repression strength constant ($GAHACR_{repstr}$) into the denominator of the hill function used to simulate the activation of GA20ox by the DELLA protein (Alon, 2006). The same constants used in the original Middleton et. al model was preserved in our model. The term GAHACR represents the abundance of the GAHACR protein in the cell, which was modelled at a range of concentrations (see Fig 1).

$$\frac{d[\mathrm{GA20ox}_m]}{d\mathrm{t}} = \varphi\mathrm{GA20ox_m}\left(\frac{[\mathrm{DELLA}]}{\left([\mathrm{DELLA}] + \theta_{\mathrm{GA20oxm}} + \left(\mathrm{GAHACR_{repstr}} \times [\mathrm{GAHACR}]\right)\right) - [\mathrm{GA20ox_m}]}\right) \quad \textbf{[11]}$$
