# Supplementary material for: Reprogramming feedback strength in gibberellin biosynthesis highlights conditional regulation by the circadian clock and carbon dioxide
Source: PLoS One. 2025 Dec 9;20(12):e0337439. doi: 10.1371/journal.pone.0337439 (PMC12688126; doi:10.1371/journal.pone.0337439)
Supplement: Attachment — Submitted filename: 20251012_GAHACR_Response to reviews.pdf [file pone.0337439.s012.pdf]

Response to reviews

Reviewer #1 (R1):
The manuscript of Nemhauser and co-authors is a systems biology study focused on understanding/manipulating gibberellin (GA) homeostasis in Arabidopsis through bioengineering. The authors (previously) constructed a GA-susceptible repressor (GAHACR) and now use it to interfere with GA signalling, correlating results with the phenotypic output. Most important is that the study is mathematical model-driven. The text is well-written, results are statistically sound, and conclusions are based on the experimental data and are fair and convincing. The work and the manuscript are perfectly suitable for the PLOS ONE scope and can be published as an article.
I suggest only minor improvements that would improve the manuscript's readability and expand the audience covered.

Our response: We thank the reviewer for their support.

R1: First, it would be better to use nomenclature/abbreviations consistently, italicise the gene and species names, remove capitalisations, place space uniformly before "ppm", and use a subscript with CO2 and Log2. Please change the acknowledgement to Dr. Imazumi, specifying "luminescence measurements" instead of "luciferase imaging".

Our response: We have made all of the suggested changes to the manuscript. Specifically, regarding nomenclature we use GAHACR in capitalized form to match the usage in our previous publication (eLife 2018). Following standard Arabidopsis nomenclature, genes are capitalized and italicized (*e.g.*, *GAI1*), proteins are capitalized and not italicized (*e.g.,* GAI1). We have endeavored to ensure this is true throughout the manuscript.

R1: It would be beneficial to display the GA chemical structure, e.g., in Fig.1, and merge panels D and E into a single panel. Additionally, the scheme in Fig. 2 Supp 1 is more visually appealing than Fig. 1B. In panel E, please replace "Without GAHACR" with "Wild type".

Our response: Thank you for your suggestions. We have included the GA4 structure in Fig. 1B. As our paper does not go into depth on the chemistry of the GA biosynthetic pathway, we hope that our inclusion of several in-depth reviews of the chemistry is sufficient for interested readers. We have adapted the text and the panel order as requested. For simplicity and compactness of the figure, we have left the cartoons of the repressors as they are.

R1: Please comment (Fig.3G and 3H and text in the results/discussion) not only on the "Days to Flowering" impact of the elevated CO2, but also how the CO2 level impacts the primary root length - it seems to be promoted.

Our response: We agree that we see an increased root length under supplemental carbon dioxide applications, and this has been seen in previous studies of carbon dioxide seeding experiments - reviewed well in 10.1042/BCJ20220245. We have

commented on this in the results section and the discussion, added this citation, and hope that readers will be interested in why the flowering time is rescued independently from the root growth phenotypes.

R1: Although it is referenced and available on github, the model would be better to describe in more details (formula, not the code) in the Methods and/or supplementary section.

Our response: We have provided the original model, descriptions of terms, and our new term in the supplemental material.

Reviewer 1: Finally, a comment is advisable on comparing GA-metabolism reprogramming and potential CO2 level impact not only in eudicots, but also in monocots. Are they not expected to be different? If so, Arabidopsis/eudicots specification should be added to the manuscript title.

Our response: The reviewer brings up a very important point, that in our opinion has not been directly investigated in the literature. It is unclear if CO2 levels will alter monocots in the same way as has been documented for dicots, and whether a similar feedback network will exist. We added additional language to the discussion to directly address this concern, which we hope monocot specialists will take note of, and therefore test directly in their systems, especially for the well described GA modulated crops in wheat and rice (such as sd1 in rice and Rht-B1b/D1b in wheat). As the title is already quite long, we have added the species name into the abstract, so that readers will know the study was done in Arabidopsis (eudicot).

--

Reviewer #2: Dear authors, you mention an ANOVA and present a heat map; however, I believe that, for the development of this work, more robust statistical analyses could be applied, which would enhance the discussion. Examples include RDA or machine learning approaches such as random forest or LDA, in order to make better use of the data. The statistical analysis you presented appears rather simplistic.

Our response: We understand that there is confusion about the statistical methods used to generate the data in figure 3. The only place where data was analyzed by ANOVA is in the statistical comparison of seedlings grown with and without supplemental carbon dioxide, and is only mentioned in the figure legend with respect to panels G & H. The RNA seq data was analyzed using DESeq2 (DOI: 10.18129/B9.bioc.DESeq2) and EdgeR (DOI: 10.18129/B9.bioc.edgeR) packages, which are considered standard in the field, and is diagrammed in the flowchart in figure 3A. We have added additional text to clarify how all data were analyzed.
